# Draft Genome Sequences of the Black Rock Fungus *Knufia petricola* and Its Spontaneous Nonmelanized Mutant

**DOI:** 10.1128/genomeA.01242-17

**Published:** 2017-11-02

**Authors:** Donatella Tesei, Hakim Tafer, Caroline Poyntner, Guadalupe Piñar, Ksenija Lopandic, Katja Sterflinger

**Affiliations:** VIBT-Extremophile Center, University of Natural Resources and Life Sciences, Vienna, Austria

## Abstract

The fungal genus *Knufia* mostly comprises extremotolerant species from environmental sources, especially rock surfaces. The draft genome sequence of the rock fungus *Knufia petricola* presented here is the first whole-genome sequence of the only species among black fungi known to have a nonmelanized spontaneous mutant.

## GENOME ANNOUNCEMENT

*Knufia petricola* (synonym, *K. chersonesos*) is a representative of black fungi, a morphological group of ascomycetes whose prominent features include melanin pigmentation, lack of recognizable sporulation, and a high stress resilience. The genus *Knufia*, order *Chaetothyriales*, family *Trichomeriaceae*, is a relatively small clade prevalently accommodating extremotolerant fungi inhabiting bare rock surfaces. Species from plants, insects, and clinical samples have been additionally reported (1–3).

*K. petricola*, originally isolated from marble in Crimea (2), presents a distribution ranging from the Mediterranean area to the Arctic (4–6). Recent advances in the study of this nonpathogenic rock-inhabiting fungus revealed a promising set of features that suggested its choice as a model organism to elucidate its rock lifestyle as well as stress survival (7). Along with the thermo- and desiccation tolerance (4), the ability of the fungus to withstand ozone levels far beyond the values considered harmful for animal and plant tissues has been shown (D. Tesei and K. Sterflinger, unpublished data). A study of the nutritional physiology of *Sarcinomyces petricola*, considered to be *K. petricola* conspecific (2), additionally demonstrated its aptitude to tolerate and grow on monoaromatic compounds (8). Furthermore, the ability to degrade aliphatic-aromatic copolyesters (D. Tesei, F. Quartinello, D. Ribitsch, G. Gübitz, and K. Sterflinger, unpublished data) makes *K. petricola* a good candidate for biodegradation and a species of biotechnological interest. Being the only species among black fungi known to have a nonmelanized pink mutant that spontaneously originated under laboratory conditions, *K. petricola* can also aid studies of melanin function, which is thus far considered to be the main protective factor in fungi (9, 10). Thus, the genome sequences of *K. petricola* are an important basis for (i) an understanding of possible biochemical pathways involved in stress resistance and polymer degradations and (ii) the characterization of the mutation-determining differences at the phenotypic level and most likely concerning a deficiency in the melanin synthesis.

Genomic DNA was isolated from the melanized and nonmelanized *K. petricola* (strains MA 5789 and MA5790, respectively, from red sandstone; Ny London, Svalbard, Norway) grown on 2% malt extract agar using a cetyltrimethylammonium bromide (CTAB)-based protocol. The elimination of melanin from DNA was performed by two phenol-chloroform purification steps. Genome sequencing was carried out using the Ion Torrent technology (Ion PGM Hi-Q View kit; Life Technologies, Inc., Carlsbad, CA, USA), according to instructions of the manufacturers. In the case of the *K. petricola* wild type (i.e., the melanized strain), a total of 1.87 Gb, with a median read length of 340 bp resulting in ~61.0× coverage, was generated and assembled with Newbler version 2.9 into a 27,759,230-bp genome containing 388 contigs, with an *N*_50_ of 265,218 bp. A total of 1.84 Gb, with a median read length of 331 bp resulting in ~59.0× coverage, was generated instead from the nonmelanized strain. The assembly consisted of 510 contigs, with an *N*_50_ of 174,743 bp and a total size of 27,731,162 bp.

### Accession number(s).

The whole-genome shotgun projects of *K. petricola* MA 5789 and MA5790 have been deposited at DDBJ/EMBL/GenBank under the accession numbers NMUC00000000 and NMQP00000000, respectively. The versions described in this paper are NMUC01000000 and NMQP01000000, respectively.
